# Pyogenic Liver Abscess with No Predisposing Risk Factors

**DOI:** 10.1155/2018/9509356

**Published:** 2018-09-04

**Authors:** Matthew Chadwick, Leonid Shamban, Michael Neumann

**Affiliations:** ^1^Department of Internal Medicine, Genesys Regional Medical Center, 1 Genesys Pkwy, Grand Blanc, MI 48439, USA; ^2^Department of Gastroenterology, Genesys Regional Medical Center, 1 Genesys Pkwy, Grand Blanc, MI 48439, USA

## Abstract

Pyogenic liver abscesses (PLA) are an uncommon cause of hospitalization in the United States. The majority of such cases are polymicrobial and are most commonly caused by seeding of infection from the biliary system. PLA is frequently associated with specific comorbidities such as diabetes mellitus, history of liver transplant, underlying hepatobiliary, or pancreatic disease. Herein, we describe a 47-year-old healthy male with no known risk factors associated with PLA who presented to the hospital with acute fever, abdominal pain, and dark colored urine. Initially the patient had a negative right upper quadrant ultrasound. However, the patient continued to have persistent fevers and abnormal liver biochemistries with negative liver serology that led to checking a magnetic resonance cholangiopancreatography which suggested multiple liver abscesses. Computer tomography guided aspiration revealed a monobacterial* Streptococcus* species within the abscess, which is commonly associated with arterial bacteremia as a source of PLA. Arterial bacteremia is one of most rare causes of PLA. The patient's septic workup was negative for any source of infection. This case demonstrates a patient with no risk factors who was diagnosed with PLA caused by apparent arterial bacteremia with no clear source of infection.

## 1. Introduction

Commonly the identifiable causes of pyogenic liver abscesses (PLA) have been attributed to hematogenous seeding, biliary, or contiguous spread [1]. Previous reports suggest that biliary spread has been associated with* E*.* coli*, portal vein seeding has been associated with* Streptococcus anginosus*, and cryptogenic causes have been associated with* Klebsiella* [2]. Certain risk factors including diabetes, underlying hepatobiliary or pancreatic disease, history of liver transplant, and chronic use of proton-pump inhibitors (PPI) are typically present in patients with pyogenic hepatic abscesses [3]. Herein we describe a case of cryptogenic streptococcus pyogenic liver abscess in an otherwise healthy male with no risk factors.

## 2. Case Presentation

A 43-year-old Caucasian male with a past medical history significant for depression which was well controlled with Prozac presented to the Emergency Department with nausea, vomiting, myalgias, and dark colored urine. He admitted to recent travel to Virginia Beach but denied any history of travel outside of the United States. He denied any high risk sexual behavior, previous blood transfusions, or intravenous (IV) drug use. He worked as a police officer with limited field work exposure. He denied any new medications including antibiotics; acetaminophen containing products; nonsteroidal medications; or over-the-counter herbal medications, vitamins, or supplements. He had no previous surgical history or dental procedures and family history was unremarkable for chronic liver disease or gastrointestinal (GI) pathology. His review of systems was negative except for subjective fevers, abdominal pain, diarrhea, and the aforementioned symptoms.

He was evaluated in the Emergency Department and was found to be tachycardic, tachypneic, and febrile with a maximum temperature of 103.1°F. Physical exam revealed a well appearing Caucasian male in no distress who was alert and oriented to place, person, and time. He was noted to have scleral icterus. Cardiovascular exam revealed tachycardia with no murmurs, rubs, or gallops. Pulmonary exam was clear to auscultation without any rales, rhonchi, or wheezing. Abdominal exam revealed a soft, nontender, nondistended abdomen with positive bowel sounds without palpable ascites or organomegaly. Dermatological exam revealed jaundice but no erythema or wounds. Examination of the extremities revealed no evidence of edema, nail splinter hemorrhages, palmar Janeway lesions, or palmar Osler nodes. Initial laboratory evaluation revealed elevated liver biochemistries with an AST of 45 U/L, ALT of 93 U/L, alkaline phosphatase of 139 U/L, total bilirubin of 5.4 mg/dL, direct bilirubin of 2.7 mg/dL, white blood cell count of 15.1 K/cmm, hemoglobin of 14.1 g/dL, platelets of 240 K/cmm, and an INR of 1.50. The patient's alkaline phosphatase was only mildly elevated; therefore the decision was made not to order a gamma-glutamyltransferase. Patient was started on empiric ceftriaxone and admitted to the hospital.

**Figure 1:**
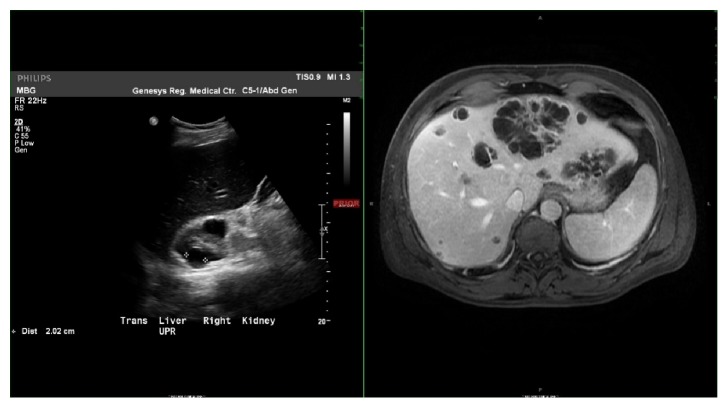
Transverse imaging showing initial negative ultrasound (left) and follow-up MRCP showing liver abscesses (right).

**Figure 2:**
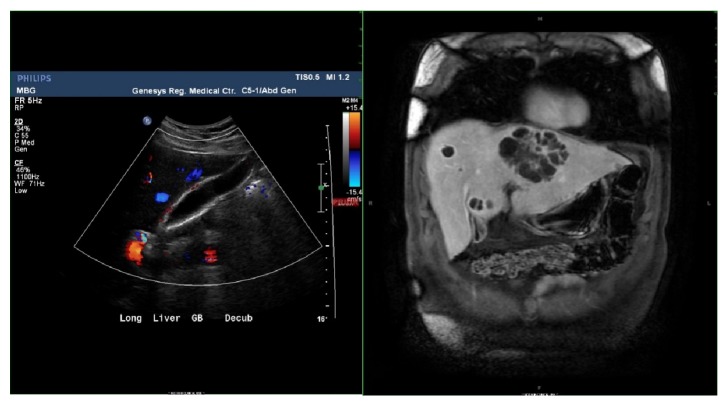
Long imaging showing initial negative ultrasound (left) and follow-up coronal MRCP showing liver abscesses (right).

Follow-up evaluation included a viral hepatitis panel (Table 1). A right upper quadrant abdominal ultrasound with Doppler flow was performed which showed a normal appearing liver without suspicious mass or biliary duct dilation, an unremarkable gallbladder, a 4 mm common bile duct, and an unremarkable liver Doppler interrogation (Figures 1 and 2).

The patient continued to have persistent symptoms of myalgias, fevers, and jaundice; therefore the gastroenterology service was consulted to evaluate the patient. Additional liver serology revealed an acetaminophen level of <5 mcg/mL, salicylate level <0.3 mg/dL, and a ceruloplasmin of 41 mg/dL, and iron studies revealed an iron level of 16 *μ*g/dl, total iron binding capacity of 171 *μ*g/dl, iron saturation of 9.4%, and a ferritin of 1914.0 ng/mL, which were consistent with inflammatory process. His autoimmune workup revealed negative smooth muscle and antimitochondrial antibodies with an ANA of <1:40.

At the same time the Infectious Disease (ID) service was consulted and a full septic workup was pursued including the workup noted in Table 1. Doxycycline was added to the regimen until chlamydia was ruled out. The patient continued to have persistent fevers and elevated liver biochemistries with an AST of 51 U/L, ALT of 61 U/L, alkaline phosphatase of 136 U/L, total bilirubin of 3.5 mg/dl, and an INR of 1.36 and therefore on his fourth day in the hospital a magnetic resonance cholangiopancreatography (MRCP) was performed which showed abnormal liver lesions concerning for multiple abscesses given the rapid change from the recent ultrasound.

Interventional radiology was consulted and using computerized tomography (CT) the largest abscess was able to be drained and produced foul-smelling turbid yellow fluid. The cultures revealed streptococcus, not group D, although the lab noted that they were not able to provide susceptibility due to the fastidious nature of the organism. Two sets of blood cultures were obtained and were negative at 120 hours. Due to the negative blood cultures and lack of physical exam findings consistent with endocarditis the ID service determined that echocardiogram was not necessary. The patient began to improve clinically, a PICC line was inserted, and the patient was discharged home on ceftriaxone. Two months later the patient had a follow-up CT of the abdomen and pelvis with and without IV contrast which showed improvement in the liver abscesses and an otherwise unremarkable liver, gallbladder, and biliary tree. Four months later the patient had a colonoscopy performed which showed no evidence of diverticular disease.

## 3. Discussion

PLA is estimated to have an annual incidence of 2.3 cases per 100,000 in the general population with a predominance of cases in males, with male: female ratio 1.3:1 [4, 5]. It is even more common amongst hospitalized patients, with one review showing an incidence of 8-22 cases per 100,000 hospital admissions [6]. PLA is the most common visceral abscess in the United States and accounts for nearly 48% of all visceral abscesses [7]. It is important to identify and treat these cases early as untreated PLA has been found to be unvaryingly fatal [8]. Even with treatment studies have found PLA to be fatal in 7.8 to 28.6% of cases [6].

Due to its fatality it is important to identify patients who may be at higher risk for developing PLA in order to aid in making a diagnosis. Several concomitant comorbid conditions have been associated with PLA including diabetes mellitus, biliary disease, hypertension, intra-abdominal infection, immunosuppression, pancreatic disease, liver transplant, malignant stricture, and inflammation of the GI tract [1, 7, 9, 10]. In the described case the patient did not have a history of hypertension, diabetes mellitus, liver transplant, or immunosuppression. The patient was only 43 years of age at the time of diagnosis, far younger than the mean age of 64 [2]. He also had extensive abdominal imaging including ultrasound, MRCP, and CT scan which did not reveal any evidence of biliary disease, pancreatic disease, malignant stricture, or inflammation in the GI tract. Studies have also shown an increased risk of PLA with PPI use [3]; however the patient had not been prescribed or taking over-the-counter PPI prior to his hospitalization.

The patient originally presented with nausea, vomiting, myalgia, and dark colored urine to the Emergency Department. The classic triad associated with PLA is compromised of fever, jaundice, and right upper quadrant abdominal pain although studies have shown that only 10% of patients with PLA present with all three symptoms [7]. More recently studies have shown that fever, chills, and abdominal pain are the three most common presenting symptoms of PLA [6, 11]. This patient did not demonstrate signs of abdominal discomfort or jaundice, although he did have persistent fevers throughout his hospital course.

In addition to these presenting symptoms the patient also had dark colored urine. While not classically associated with PLA there have been case reports of patients presenting with dark colored urine in a patient with PLA caused by* Klebsiella pneumonia* [4], while our patient's cultures were consistent with* Streptococcus*. To our knowledge there have been no reported cases of dark colored urine presenting from a patient with PLA caused by a* Streptococcus* species. The patient did also present with mildly elevated liver transaminases, which correlates with previous reports that streptococcus anginosus group often has less pronounced liver transaminases compared to other causes of PLA [12].

Previously appendicitis was the most common cause of PLA; however due to improved detection and treatment of appendicitis this is no longer a common cause of PLA [8]. The most common causes of PLA are biliary tract disease, portal bacteremia, contiguous spread of infection from intra-abdominal pathology, and intestinal disease [1, 3, 13]. Of these sources biliary tract disease has been found to be the most common cause of PLA in the United States, causing an estimated 50-60% of all cases [1, 8]. Portal bacteremia is the second most common cause of PLA accounting for 10-20% of all cases of PLA [1]. The most common cause of portal bacteremia is complicated sigmoid diverticulitis, with less common causes being supra-infected GI tumors and inflammatory bowel disease [1]. After biliary disease, perforated bowel has been found to be the next most common intra-abdominal cause of PLA formation [1, 14, 15]. Again, despite these being the most common cause of PLA our patient's lab work and imaging showed no evidence of biliary or other intra-abdominal pathology. It is noted however that abdominal ultrasound depends on the experience of the operator and may miss a slight dilation of the bile duct or small calculi in the bile duct in its retropancreatic portion. He also had blood cultures that were negative.

Another cause of PLA is arterial hematogenous spread, although this has been found to be a very rare cause [8, 9, 14]. The most common cause of arterial bacteremia leading to PLA formation is endocarditis, pyelonephritis, lung infections, peripheral suppurative thrombophlebitis in IV drug users, and oral infections [1, 8, 9]. These cases are usually found in patients with concomitant diabetes mellitus, leukemia, immunosuppression, or chronic granulomatous disease [1, 8]. There were no clinical signs of pulmonary, oral, or genitourinary infection during the patient's hospitalization. The patient denied any history of IV drug abuse and no clinical signs of thrombophlebitis were noted during his hospital stay. The patient did also have negative blood cultures and was determined by the ID team to be at minimal risk for infective endocarditis. In addition, there was no clinical evidence to suggest that the patient had diabetes mellitus, leukemia, immunosuppression, or chronic granulomatous disease.

The majority of cases of PLA are polymicrobial [3, 7, 14]. Monomicrobial PLA is more commonly associated with hematogenous spread, especially arterial origin [1, 7]. Studies have found that the majority of PLA from arterial bacteremia have positive blood cultures. Also 66% of PLA patients have positive blood cultures regardless of its origin [1, 6]. The three most common bacteria to cause PLA are* E*.* coli*,* Klebsiella pneumonia*, and* Streptococcus* species [7]. The patient in the above described case had a single organism, streptococcus not group D, cultured from his abscess. Monobacterial PLA, especially from* Streptococcus* species, is highly indicative of hematogenous spread [8]. Our patient had no evidence of bacteremia, no identifiable sources for bacteremia, and no evidence of concomitant comorbidities associated with arterial bacteremia known to cause PLA.

## Figures and Tables

**Table 1:** Infectious disease workup.

Test	Results
Hepatitis A IgM	Nonreactive
Hepatitis B Surface Antigen	Nonreactive
Hepatitis B Core Antibody IgM	Nonreactive
Hepatitis C Antibody	Nonreactive
Hepatitis C Viral RNA	<30 IU/mL
Influenza A	Negative
Influenza B	Negative
Urine Culture	No growth
Epstein Barr Viral Antibody IgG	137.0 U/ml
Epstein Barr Viral Antibody IgM	<10.0 U/ml
Cytomegalovirus Antibody IgG	1.50 U/ml
Cytomegalovirus Antibody IgM	<8.0 AU/mL
N. Gonorrhea PCR	Not detected
C. trachomatis PCR	Not detected
RPR	Non-reactive
Giardia Lamblia	Negative
Cryptosporidium	Negative
